# Fecal Serine Protease Profiling in Inflammatory Bowel Diseases

**DOI:** 10.3389/fcimb.2020.00021

**Published:** 2020-02-04

**Authors:** Amin Jablaoui, Aicha Kriaa, Héla Mkaouar, Nizar Akermi, Souha Soussou, Magdalena Wysocka, Dominika Wołoszyn, Ali Amouri, Ali Gargouri, Emmanuelle Maguin, Adam Lesner, Moez Rhimi

**Affiliations:** ^1^Université Paris-Saclay, INRAE, AgroParisTech, Micalis Institute, Microbiota Interaction with Human and Animal Team (MIHA), Jouy-en-Josas, France; ^2^Faculty of Chemistry, University of Gdansk, Gdansk, Poland; ^3^Department of Gastroenterology, Hedi Chaker University Hospital, Sfax, Tunisia; ^4^Laboratory of Molecular Biology of Eukaryotes, Center of Biotechnology of Sfax, University of Sfax, Sfax, Tunisia

**Keywords:** fecal proteases, inflammatory bowel diseases, gut microbiota, protease profiling, serine protease inhibitors, Holobiont, microbiome

## Abstract

Serine proteases are extensively known to play key roles in many physiological processes. However, their dysregulation is often associated to several diseases including inflammatory bowel diseases (IBD). Here, we used specific substrates to monitor fecal protease activities in a large cohort of healthy and IBD patients. Of interest, serine protease activity was 10-fold higher in IBD fecal samples compared to healthy controls. Moreover, functional analysis of these fecal proteolytic activities revealed that the most increased activities are trypsin-like, elastase-like and cathepsin G-like. We also show for the first time, an increase of proteinase 3-like activity in these samples compared to controls. Results presented here will guide further investigations to better understand the relevance of these peptidases in IBD.

## Introduction

Over one-third of known peptidases are serine proteases (Hedstrom, 2002). They are involved in a plethora of biological processes including digestion, immune responses, and serve as essential signaling molecules in gastrointestinal physiology (Vergnolle, 2016). These proteases are often expressed at regulated levels under physiological conditions, being secreted by intestinal cells, infiltrating immune cells in the lamina propria and the gut microbiota (Biancheri et al., 2013). Serine protease activity is tightly regulated and the disequilibrium of this proteolytic activity is linked to several gastrointestinal disorders including inflammatory bowel diseases (IBD) which constitute a worldwide problem with a high incidence (Kaplan, 2015; Edgington-Mitchell, 2016; Masaki et al., 2018). An increased expression level of host serine proteases was previously observed in colonic samples from IBD patients (Raithel et al., 2001; Dabek et al., 2009; Motta et al., 2012; Hamilton et al., 2014). Such dysregulated proteolysis was further described to elicit structural and functional changes in the mucosal barrier and participate in inflammation (Van Spaendonk et al., 2017). Surprisingly, most studies addressing the proteolytic balance in this context have analyzed expression profiles while largely ignoring to take in account their activity levels. Further *in situ* assays revealed an increased proteolytic activity within the epithelium during inflammation (Rolland-Fourcade et al., 2017). Nevertheless, this activity was mainly ascribed to epithelial serine proteases and infiltrating immune cell peptidases as human neutrophil elastase, cathepsin G, tryptase, chymase, trypsin, etc… (Devaney et al., 2003; Sun et al., 2004; Lefrancais et al., 2012). The identity of upregulated serine proteases remains unknown and their potential contribution to the overall luminal proteolysis is unclear. The emergence of new systems biology technologies has helped to monitor active serine proteases in health and disease (Pan et al., 2006; Poulsen et al., 2012; Starr et al., 2017). Using functional proteomic assays via activity-based probes, different profiles of active serine proteases were detected in colonic biopsies from patients with Crohn's disease (CD) and ulcerative colitis (UC) (Denadai-Souza et al., 2018). However, the role of protease activity encoded by the gut microbiota remains poorly studied. The development of new tools to characterize protease activities in fecal samples constitutes a challenge to investigate this activity. Therefore, the use of substrates for different protease families appears as an efficient approach to understand their distribution in healthy and IBD subjects.

In this report, we demonstrate that protease activity is increased in IBD patients compared to healthy subjects. Analysis of these protease activities showed that serine protease family constitutes the most active protease family. Furthermore, we proved that trypsin, neutrophil elastase (HNE), proteinase 3 (PR3), and cathepsin G (CatG) were most dominant among other serine proteases.

## Materials and Methods

### Study Participants and Fecal Sample Collection

Demographic data of patients associated to this work are shown in Table 1. The study group consists of 50 IBD patients (25 CD, 25 UC) and 50 healthy subjects. Fecal samples were collected from patients in the region of Sfax (Tunisia) at the Department of Gastroenterology of the hospital Hedi Chaker (Sfax-Tunisia). All participants were subjected to a clinical examination and an analysis of their medical history including mainly (i) no treatment against IBD or other diseases before, (ii) determination of the inflammatory profile, and (iii) diagnosis by radiographic studies and endoscopy. Individuals having antibiotic or anti-inflammatory treatment during the last 6 months were excluded. The ethical committee of CHU Hedi Chaker (Sfax-Tunisia) approved our protocol (Authorization number: CPP SUD No. 0203/2019). Fecal samples were collected from each subject and rapidly stored at −80°C until activity monitoring.

**Table 1:** Demographic data.

**Patient groups**	**Number**	**Gender**	**Age mean (SEM)**
Healthy	50	24 F/26 M	39.16 (2.02)
CD	25	11 F/14 M	34.4 (1.98)
UC	25	10 F/15 M	31.5 (1.70)

### Fecal Water Sample Preparation

For each biological sample, 1 g of frozen feces was thawed and homogenized in 5 ml of Tris-HCl buffer (0.2 M NaCl, Tris-HCl 20 mM pH 7.8). After centrifugation (15 min, 5,000 rpm, 4°C), pellets were discarded and supernatants were filtered using size syringe filters (0.8 μm, Nalgene). Obtained supernatants were then used for protease activity measurements.

### Protease Activity Assays

Protease activity was measured by estimating the amount of chromogenic/fluorogenic compounds released after proteolytic cleavage. Under standard conditions, the reaction mixture containing 20 μl of fecal water sample at a suitable dilution (100 μg), 160 μl of reaction buffer (0.2 M NaCl and 20 mM Tris-HCl, pH 7.8), and 20 μl of the appropriate substrate to a final volume of 200 μl was incubated during 30 min at 37°C. For azocasein, the reaction was stopped by adding 100 μl of 10% (w/v) trichloroacetic acid (TCA, Sigma). For protease activity profiling, we used different substrates listed in Table 2. Activity measurements were performed with chromogenic (410 nm) and fluorogenic (Excitation: 360 nm, Emission: 460 nm) substrates using a plate reader (Perkinelmer) at room temperature. For inhibition assays, we used selective protease inhibitors, PMSF (Phenylmethylsulfonyl fluoride, 1 mM), SBTI (soybean trypsin inhibitor, 0.5 mM), E-64 (10 μM), Trypsin inhibitor (100 μM), HNE inhibitor (10 μM) (sigma), PR3 inhibitor (0.1 μM), Human neutrophil elastase inhibitor (Sigma, 0.1 μM), and CatG inhibitor (10 μM) (Table 2). Protein concentration was assessed using Nanodrop (Labtech) at 280 nm (1 abs = 1 mg/ml). One unit of protease activity was defined as the amount of enzyme catalyzing the formation of 1 μmol of substrate per min under the above experimental conditions.

**Table 2:** Serine protease specific substrates and inhibitors.

**Substrate/** **inhibitor**	**Sequence**
Trypsin-like	AB2-Val-Val-Ser-Lys-ANB-NH2 (Chromogenic)
HNE-like	AB2-Met-Pro-Val-Ala-Trp-Glu-Tyr-(3-NO2)-NH2 (Fluorogenic)
PR3-like	AB2-Tyr-Tyr-ABU-Asn-Glu-Pro-Tyr-(3-NO2)-NH2 (Fluorogenic)
CatG-like	MCA-Phe-Val-Thr-Gnf-Ser-Trp-AB2-NH2 (Fluorogenic)
Chymotrypsin-like	AB2-Lys-His-Trp-Tyr-ANB-NH2 (Chromogenic)
Cysteine-like	AB2-Ile-Leu-Pro-Glu-ANB-NH2 (Chromogenic)
Trypsin inhibitor	N- a-Tosyl-L-Lys Chloromethyl Ketone Hydrochloride
HNE inhibitor	N-(Methoxysuccinyl)-Ala-Ala-Pro-Val-chloromethyl ketone
PR3 inhibitor	Bt-Val-Tyr-Asp-nValP(O-C6H4-4-Cl)2
CatG inhibitor	Ac-Phe-Val-Thr-PhgP(4-guanidine)-(OC6H4-4-S-Me)2

### Casein Zymography

SDS-PAGE zymograms were performed as previously reported with minor modifications (Bencsik et al., 2017). Eight percentage of polyacrylamide gels were copolymerized with 0.1% casein. Samples were prepared in non-reducing loading buffer without heat denaturation and run at 120 V during 1 h. Following electrophoresis, the gels were washed for 1 h in 100 mM phosphate buffer pH 6.8, containing 2.5% Triton X-100 (Merck), with gentle agitation aiming the removal of the excess of SDS. Subsequently, the gels were incubated during 5 h with several changes in a solution of 100 mM phosphate buffer pH 6.8. Zones of proteolysis were revealed using Coomassie blue staining.

### Statistical Analyses

All data are shown as means ± SEM. For statistical analysis, Graph Pad Prism 7.0 (GraphPad software, Inc.) was used. Differences in fecal protease activity between controls and patients (CD & UC) were assessed using Kruskal-Wallis test followed by Dunn's test for multiple comparison test. For inhibition assays, data were analyzed using Mann Whitney test to compare the proteolytic activity with and without inhibitors. Statistical significance was accepted at *p* < 0.05.

## Results

### Protease Activity Is Upregulated in IBD Fecal Samples

**Figure 1:**
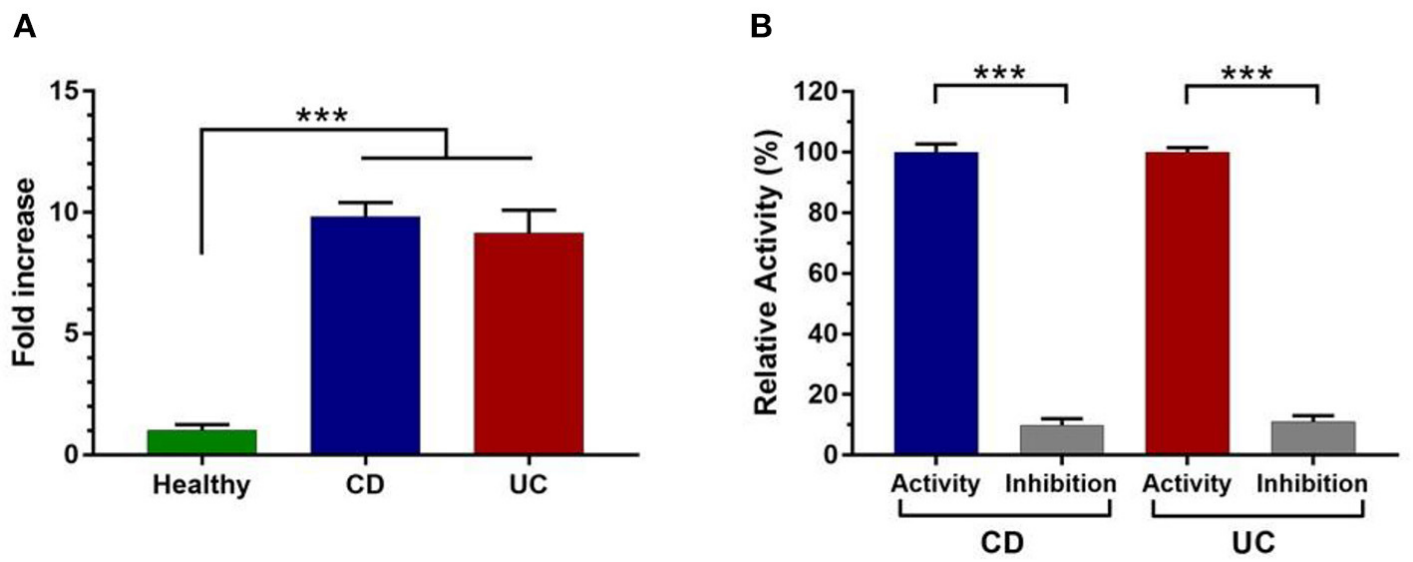
Measurement of total protease activity in fecal samples of control (*n* = 50) and IBD patients (*n* = 50). **(A)** Total fecal proteolytic activity in healthy subjects and IBD patients. **(B)** The relative proteolytic activity with or without pretreatment with PMSF in CD and UC samples. Data are mean ± SEM. Data were analyzed by Kruskal-Wallis test followed by Dunn's test. The relative activity corresponds to the maximal activity defined as 100% (CD = 363 U/mg and UC = 339 U/mg). Mann Whitney test was performed to compare the proteolytic activity without and in the presence of inhibitor (PMSF) in CD and UC patient. ***p < 0.001.

Fecal protease activity measurement demonstrates that total protease activity was 10- and 9-fold higher in patients with CD and UC, respectively, compared to healthy controls (Figure 1A). To reinforce these data, several specific protease inhibitors were tested for their impact on the detected proteolytic activities. As shown in Figure 1B, the proteolytic activity was significantly reduced by 90% in both CD and UC samples (*p* < 0.001) in presence of PMSF (Figure 1B). Considering that PMSF is a broad-spectrum serine protease inhibitor, we concluded that serine protease activity increases the most among proteases deriving from fecal water in IBD patients. Such results were confirmed by the analysis of other protease families including cysteine proteases and metalloproteases; however, no significant difference was observed between IBD and healthy subjects (Supplementary Figure 1).

### Profiling of Fecal Serine Proteases and Inhibition Assays

**Figure 2:**
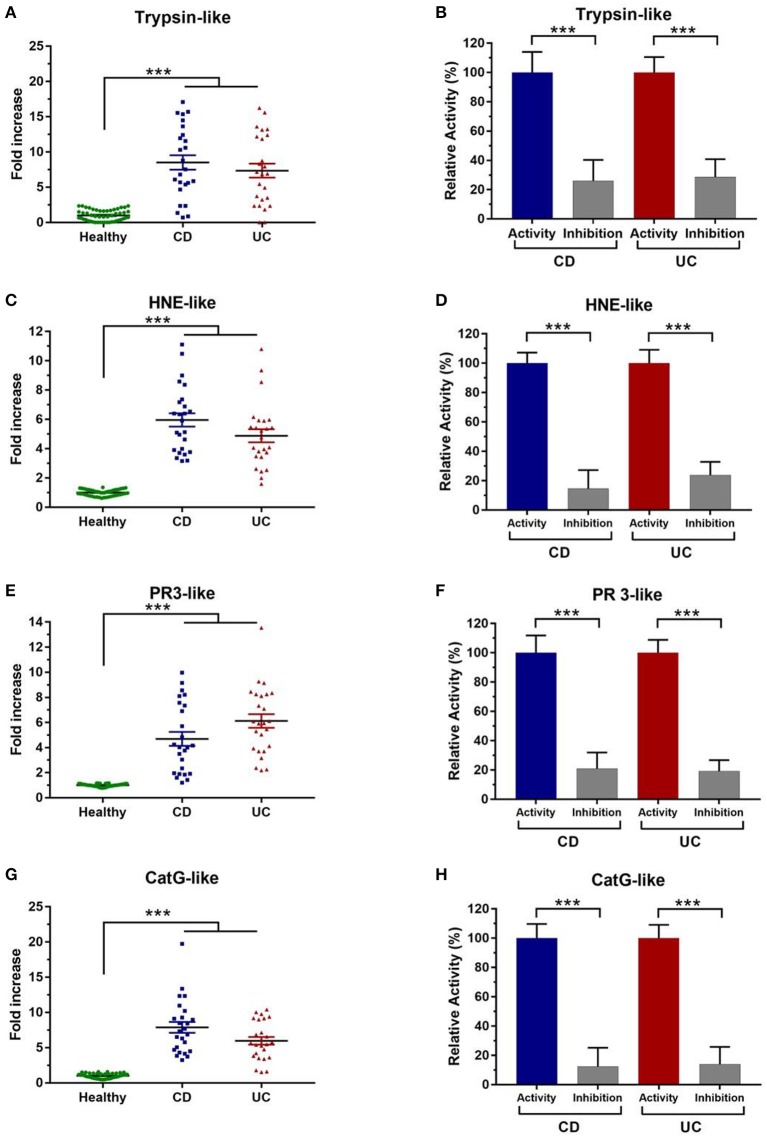
Characterization of fecal serine protease activity using specific inhibitors. **(A)** Fecal trypsin-like activity in healthy subjects (*N* = 50) and IBD patients (*N* = 50) (CD = 278 U/mg and UC = 234 U/mg). **(B)** Trypsin-like activity without and in the presence of their specific inhibitor. **(C)** Fecal HNE-like activity in healthy subjects and IBD patients (CD = 126 U/mg and UC = 105 U/mg). **(D)** HNE like-activity without and in the presence of their specific inhibitor. **(E)** Proteinase 3-like activity in healthy individuals and IBD patients (CD = 85 U/mg and UC = 102 U/mg). **(F)** Inhibition assay of PR3-like activity in the presence of their specific inhibitor. **(G)** Cathepsin G-like activity in healthy individuals and IBD patients (CD = 68 U/mg and UC = 54 U/mg). **(H)** Inhibition assay of Cat G-like activity in the presence of their specific inhibitor. The relative activity corresponds to the maximal activity defined as 100%. The error bars represent the SEM. Data were analyzed by Kruskal-Wallis test followed by Dunn's test to compare protease activity in CD and UC patient to healthy control. Mann Whitney test was performed to compare the proteolytic activity without and in the presence of each inhibitor in CD and UC patient. ***p < 0.001.

To further investigate the increased serine protease activity in IBD patients, we used specific substrates. Of interest, trypsin-like activity was 8-fold higher in IBD fecal samples than healthy individuals (*p* < 0.001) (Figure 2A). To confirm the selectivity of the designed substrates, we used a specific inhibitors designed for each targeted protease family, fecal trypsin-like activity was significantly reduced to around 26 and 28% in CD and UC samples, respectively (Figure 2B, Supplementary Figure 2A). These data clearly demonstrate that the analyzed proteolytic activities belong to trypsin-like subfamily.

Chymotrypsin-like activity was also assessed in these samples using its specific substrate. However, no detectable fecal activity was observed (data not shown). On the other hand, HNE and PR3-like activities were remarkably increased in IBD samples. In comparison with healthy subjects, samples from IBD patients showed statistically higher elastolytic activity (6- and 5-fold higher in CD and UC, respectively, *p* < 0.001) (Figure 2C). To reinforce these observations, we used a specific inhibitor for HNE, which allowed a significant decrease to around 15% in CD samples and to 23% in UC patients (*p* < 0.001) (Figure 2D, Supplementary Figure 2B). Moreover, we demonstrated that the Human Elastase inhibitor (Elafin) significantly inhibits the fecal HNE-like (Supplementary Figure 2C). Then, we sought to assess fecal PR3 activity which was significantly increased in samples from CD and UC patients (*p* < 0.001) (Figure 2E). Using its specific inhibitor, PR3-like activity was significantly inhibited in CD (21%) and in UC samples (19%) (Figure 2F). To the best of our knowledge, it is the first work aiming to assess PR3 activity and its inhibition in IBD samples. A high activity for CatG-like was also detected in IBD fecal supernatants. Indeed, fecal CatG-like activity was around 8- and 6-fold higher in samples from CD and UC patients than healthy controls (Figure 2G). Moreover, using a specific inhibitor, this activity was dramatically decreased by 88 and 86 % in CD and UC supernatants, respectively (Figure 2H, Supplementary Figure 2D). These data show that CatG is highly abundant in fecal samples of IBD patients but not in healthy controls.

## Discussion

IBD have emerged as a major concern worldwide (Kaplan, 2015). They are associated to a serious economic burden, treatments are far efficient and limited data are available regarding their pathogenesis (Masaki et al., 2018). Analysis of the gut microbiota composition including mainly increase of the Actinobacteria and Proteobacteria and a decrease of the Firmicutes and Bacteroidets phyla (Frank et al., 2007). Furthermore, proteases are largely present in the gastrointestinal tract, where they are known to be involved in the inflammatory response (Vergnolle, 2016). Several reports have demonstrated that a dysregulated proteolysis may contribute to several digestive inflammatory diseases including mainly the irritable bowel syndrome (Róka et al., 2007; Edgington-Mitchell, 2016; Vergnolle, 2016). Using human colonic mucosa, it was described the dysregulation of thrombin and Cathepsin G in IBD patients (Denadai-Souza et al., 2018). Moreover, it was reported that (i) contrary to the conventional mice, the axenic mice did not develop inflammatory disorders indicating the key role of gut microbiota on IBD and (ii) fecal supernatants from UC and CD induce an increase of permeability in mice (Dabek et al., 2009; Carding et al., 2015). In this study, we show that protease activity is significantly higher in IBD fecal samples than healthy subjects. Interestingly, among all proteolytic enzymes, serine proteases displayed the most increased activity (Figure 1). Similar increases in levels of protease activity were previously observed in fecal contents (Róka et al., 2007). In fact, using fecal samples from 15 to 17 UC patients, these studies denoted an elevated serine protease activity to nearly 3- to 4-fold, compared to controls (Róka et al., 2007; Gecse et al., 2008). Herein, we show an increased serine protease activity to 10-fold higher in IBD fecal supernatants that was then confirmed using specific inhibitors (Figure 1). Interestingly, the specific substrates and inhibitors were selected based on their specificity to target special protease family. To the best of our knowledge, this work constitutes the first study showing the highest level of serine protease activity from a large cohort of Tunisian IBD patients. To better unravel the identity of active serine proteases in these samples, we used specific substrates for each protease subfamily. Compared to control subjects, trypsin-like activity was greatly increased in fecal IBD samples (Figure 2A). These findings are consistent with other studies stressing the high level of such activity in IBD mucosal biopsies and fecal contents (Midtvedt et al., 2013; Denadai-Souza et al., 2018). In these studies, tryptic activity was around 4- and 100-fold higher in IBD colonic tissues and CD fecal samples, respectively (Midtvedt et al., 2013; Denadai-Souza et al., 2018). However, only few samples of patients were analyzed to assess proteolytic activity (Midtvedt et al., 2013; Denadai-Souza et al., 2018). HNE-like activity was also higher in CD and UC supernatants (Figure 2C). Similarly, an increased neutrophil elastase activity was reported to be 3-fold higher in fecal samples and around 100-fold higher in colonic biopsies (Bustos et al., 1998; Motta et al., 2012). Such activity was further suggested to be linked to the high proteolytic activity observed in colonic biopsies (Motta et al., 2012). Although a causal relationship between PR3 and IBD is not yet established, PR3- autoantibodies were proposed as a promising biomarker for UC (Mahler et al., 2012). Of note as illustrated in Figure 2E, PR3-like activity was significantly increased in samples from CD and UC patients compared to healthy controls (*p* < 0.001). As far as we know, this is the first work that shows a high PR3-like activity in IBD. CatG is known to be involved in intestinal inflammation (Dabek et al., 2009). Using the specific substrate and inhibitor for this protease, we demonstrate that fecal CatG-like activity is significantly higher in IBD samples (Figures 2G,H). These data support the correspondence of these pronounced activities in IBD fecal supernatants to CatG-like proteases. In this context, a previous study described a 14-fold increase of fecal CatG activity in IBD patients, which was correlated with disease severity (Dabek et al., 2009). Moreover, using functional proteomic assays through activity-based probes, active CatG was also detected in the secretome from UC colonic tissues (Denadai-Souza et al., 2018). Interestingly, it was demonstrated that upregulated protease families including Trypsin-like and CatG-like can activate PAR-2 and PAR-4, respectively (Cenac et al., 2002; Dabek et al., 2009). Moreover, it was demonstrated that proteases are linked to the increase of the intestinal permeability through their action on tight-junction proteins (Zeissig et al., 2007; Dabek et al., 2009). Interestingly study of the fecal proteases by zymographic analysis demonstrates that UC and CD subjects display different proteolytic profiles revealing again the differential distribution of the fecal proteases (Supplementary Figure 3). Such data stress the key role of these serine proteases in digestive inflammation and that a disequilibrium of such proteolytic activities is associated to these inflammatory disorders.

To summarize, this approach using rapid specific substrates to monitor fecal proteases in IBD samples allowed us to assess proteolytic activities previously reported to be increased in IBD mucosal tissues (trypsin-like, CatG-like) (Denadai-Souza et al., 2018), but also others not yet described in this setting (PR3-like activity). Besides, this is the first time that such a large Tunisian cohort is characterized and stressing the association of serine proteases to IBD using a powerful strategy of protease activity analysis. Although this study did not investigate the origin of elevated serine protease activity in these samples, both host and bacterial serine proteases with overlapping specificities may be involved. Analysis of (i) other large cohorts of IBD and healthy subjects, (ii) aging and diet on the distribution of the fecal proteases in healthy and IBD subjects, and (iii) the abundance of the genes encoding for serine proteases in human metagenomics catalog will confirm the potential of these proteases as targets to treat IBD. Interestingly, we plan to investigate the effect of proteases on other intestinal inflammatory disorders including inflammatory bowel syndrome. Furthermore, analysis of the microbial and human proteases using engineered animal models can help to decipher the impact of each. This will significantly contribute to the better understanding of the host-microbiota interplay that modulates the host well-being.

## Data Availability Statement

All datasets generated for this study are included in the article/Supplementary Material.

## Ethics Statement

The studies involving human participants were reviewed and approved by Comité d'éthique du CHU Hédi Chaker—SFAX (Tunisie). The patients/participants provided their written informed consent to participate in this study.

## Author Contributions

AJ, AK, HM, NA, AA, AG, AL, EM, and MR conceived the scientific ideas. AJ, AK, HM, NA, MW, DW, AA, SS, AL, EM, and MR performed and discussed the work and edited the manuscript. All authors reviewed the manuscript and provided critical feedbacks.

### Conflict of Interest

The authors declare that the research was conducted in the absence of any commercial or financial relationships that could be construed as a potential conflict of interest.
