# Supplementary material for: Fecal Serine Protease Profiling in Inflammatory Bowel Diseases
Source: Front Cell Infect Microbiol. 2020 Feb 4;10:21. doi: 10.3389/fcimb.2020.00021 (PMC7011180; doi:10.3389/fcimb.2020.00021)
Supplement: Supplementary file 1 [file Data_Sheet_1.docx]

Supplementary Material

## Supplementary Figure


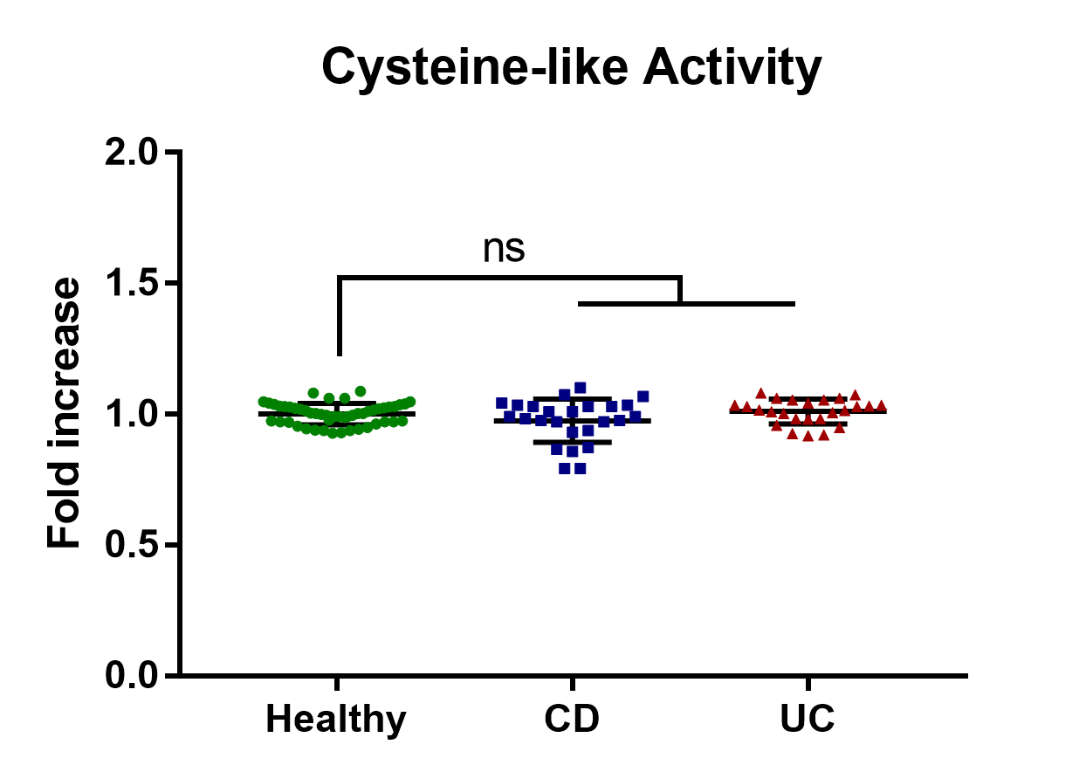


**Supplementary Figure 1.** Cysteine-like activity in healthy subjects and IBD patients (Healthy = 12.6 U/mg and, CD = 12.28 U/mg, UC= 12.73 U/mg). The error bars represent the SEM. Data were analyzed by Kruskal-Wallis test followed by Dunn’s test to compare protease activity in CD and UC patient to healthy control. ns: non-significant (p > 0.05).


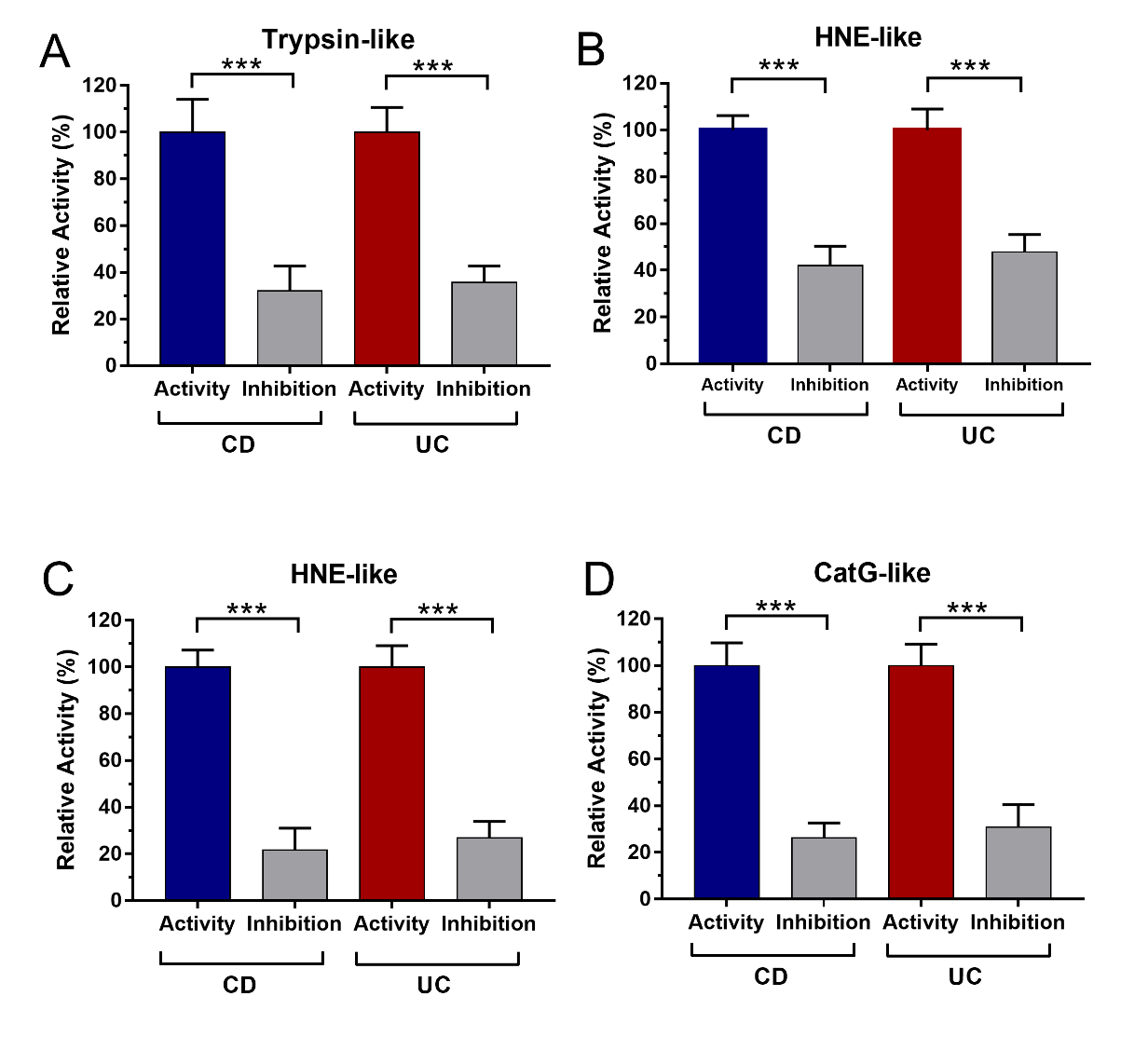


**Supplementary Figure 2.** Serine protease activity without and with the presence of specific inhibitors. (A) Trypsin-like activity without and in the presence of SBTI. The relative activity corresponds to the maximal activity defined as 100% (CD = 278 U/mg and UC = 234 U/mg). (B) HNE-like activity without and in the presence of Elafin. The relative activity corresponds to the maximal activity defined as 100% (CD = 126 U/mg and UC = 105 U/mg). (C) HNE like activity without and in the presence of elastase inhibitor II. The relative activity corresponds to the maximal activity defined as 100% (CD = 126 U/mg and UC = 105 U/mg). (D) Cathepsin G-like activity without and in the presence of cathepsin G inhibitor. The relative activity corresponds to the maximal activity defined as 100% (CD = 68 U/mg and UC = 54 U/mg). The error bars represent the SEM. Data were analyzed by Mann Whitney test to compare protease activity in CD and UC patients without and in the presence of their specific inhibitor. *** p < 0.001.


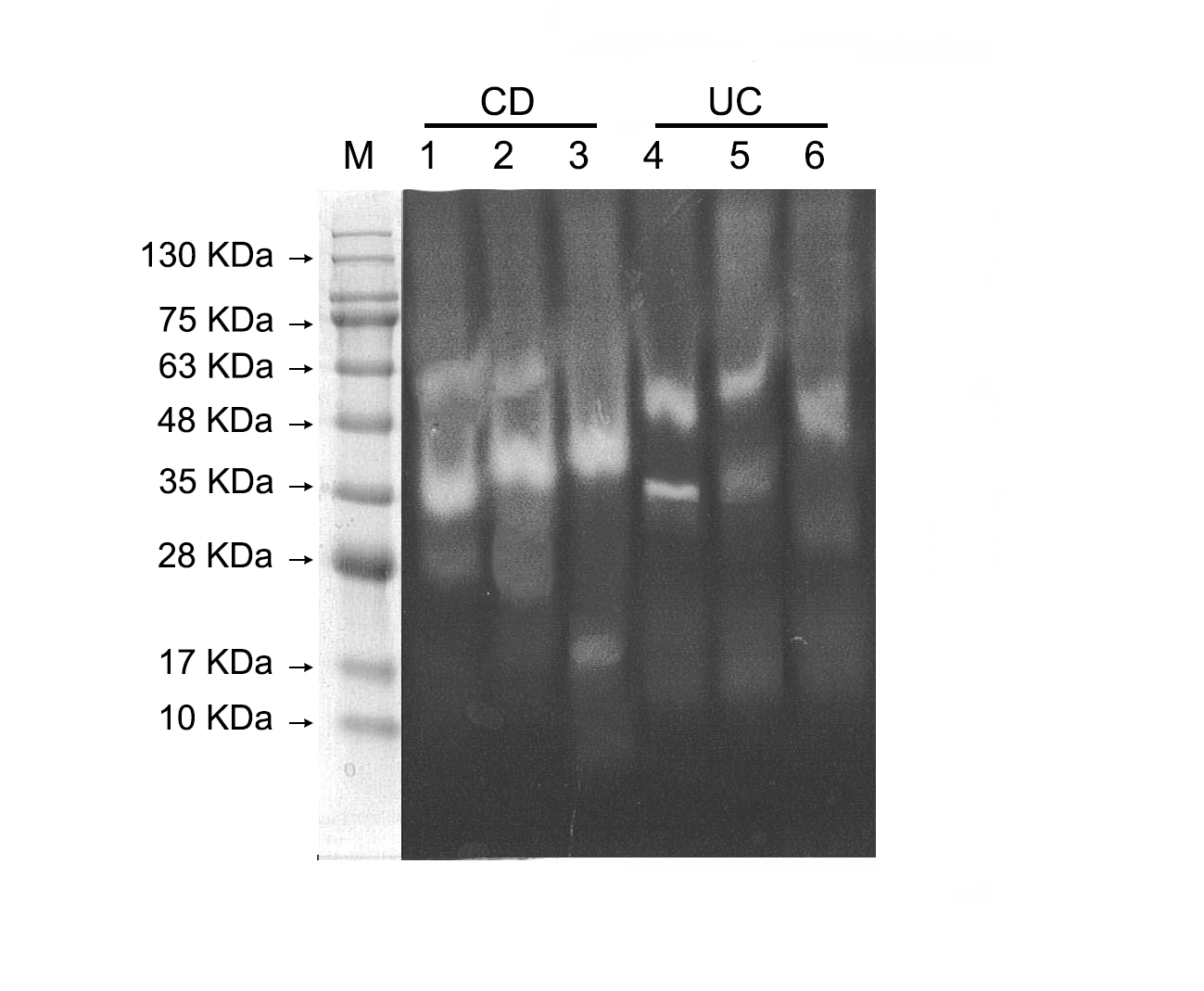


**Supplementary Figure 3.** Example of casein zymography of CD and UC Patients. Fecal supernatant from CD and UC patient (amount of 25µg) was electrophoresed into casein gel and then incubated in a proteolysis buffer. Panel M, represents a molecular protein marker (BlueStar Prestained protein marker, Genetics), wells 1-3: samples from CD patients and wells 4-6: samples from UC patients.
